# Supplementary material for: Understanding Callus Types in Maize by Genetic Mapping and Transcriptional Profiling
Source: Plants (Basel). 2025 Oct 15;14(20):3168. doi: 10.3390/plants14203168 (PMC12566775; doi:10.3390/plants14203168)
Supplement: Supplementary file 1 [file plants-14-03168-s001.zip › supplementary_files/Supplementary_figures.docx]

# Supplementary Figures

# Understanding callus types in maize by genetic mapping and transcriptional profiling

Lin et al.

**Figure S1. Detailed characterization of QTL ctAB2a**

(**A**) Genetic mapping of callus types with GBS segment markers on chromosome 2. The gray dash line indicates the LOD threshold of 3. (**B**) Genetic mapping of callus types from BSR-seq. The orange dash line indicates the threshold defined by the Bonferroni correction at the 5% significance level. The significance SNP markers are colored in green. The vertical purple dash lines (in both **A** and **B**) indicate the LOD support QTL interval, and the red vertical dash line indicates the left flanking of the interval adjusted based on the BSR-seq mapping. (**C**) The distribution of genotypes of the 60 XT-I F2 calli and 58 XT-II F2 calli on chromosome 2. The red rectangle box indicates the QTL interval, and the red triangle points at the QTL peak. (**D**) Distribution of callus types in three genotypes. AA: A188 homozygous genotype; BB: B73 homozygous genotype; AB: heterozygous genotype.


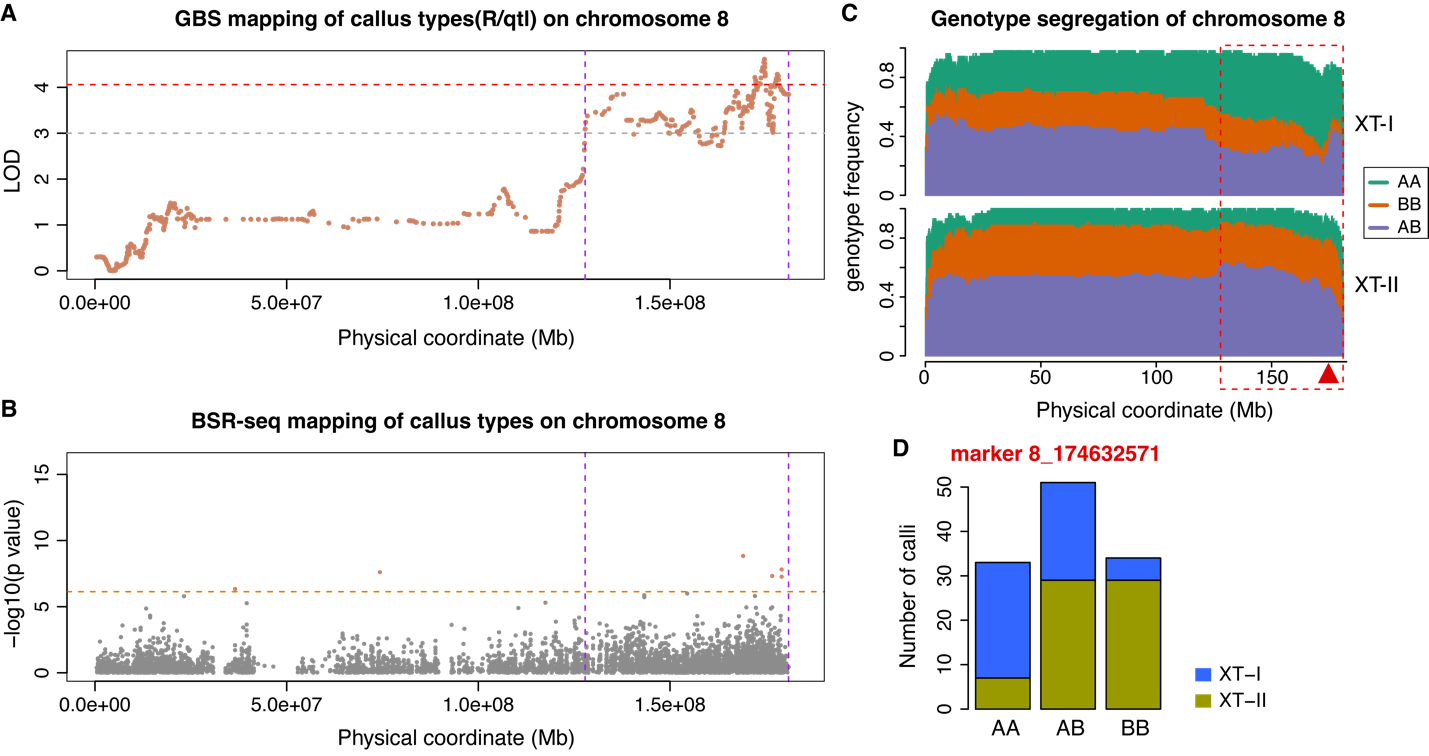


**Figure S2. Detailed characterization of QTL ctAB8a**

(**A**) Genetic mapping of callus types with GBS segment markers on chromosome 8. The red dash line indicates the significance threshold defined by permutation tests at the 5% significance level, and the gray dash line indicates the LOD threshold of 3. (**B**) Genetic mapping of callus types from BSR-seq. The orange dash line indicates the threshold defined by the Bonferroni correction at the 5% significance level. The significance SNP markers are colored in green. The vertical purple dash lines (in both **A** and **B**) indicate the LOD support QTL interval, and the red vertical dash line indicates the left flanking of the interval adjusted based on the BSR-seq mapping. (**C**) The distribution of genotypes of the 60 XT-I F2 calli and 58 XT-II F2 calli on chromosome 8. The red rectangle box indicates the QTL interval, and the red triangle points at the QTL peak. (**D**) Distribution of callus types in three genotypes. AA: A188 homozygous genotype; BB: B73 homozygous genotype; AB: heterozygous genotype.


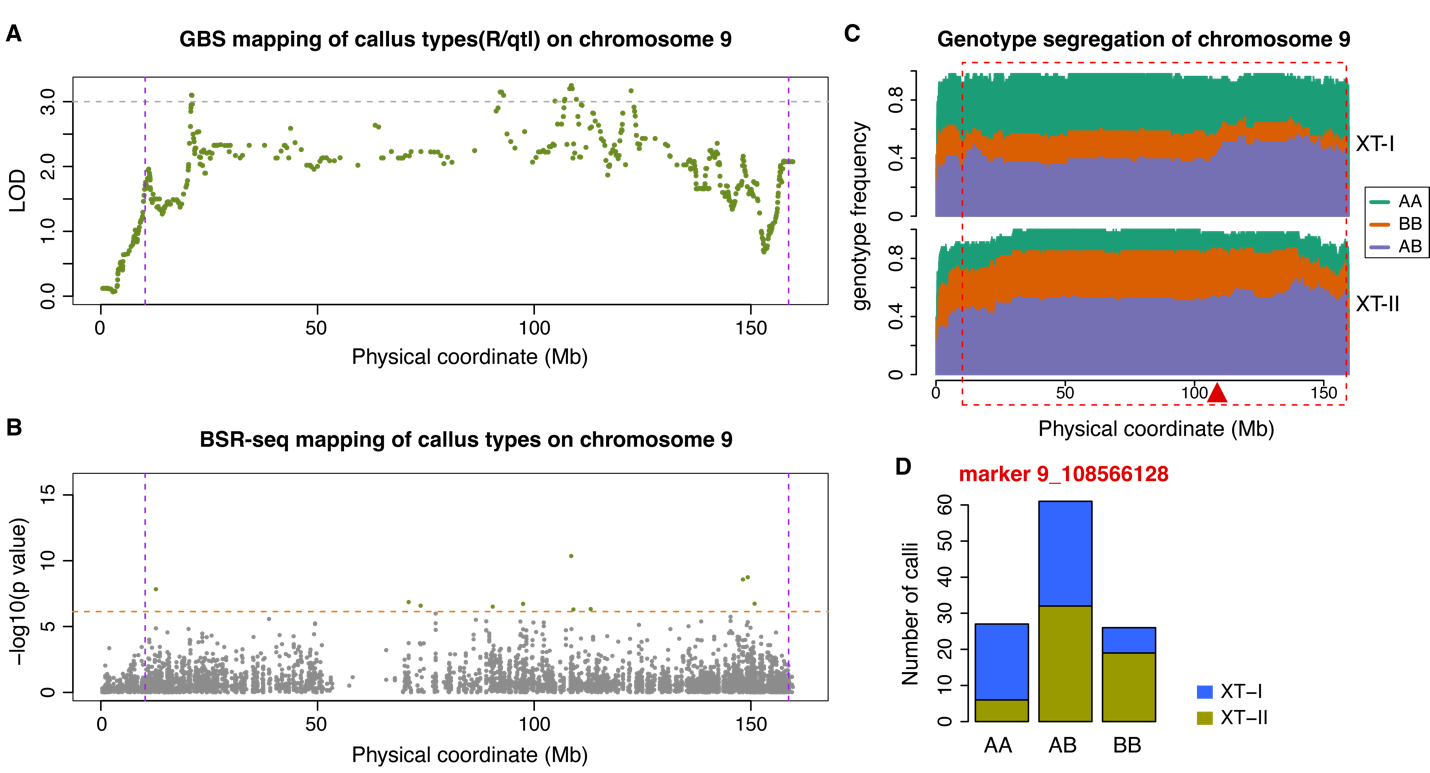


**Figure S3. Detailed characterization of QTL ctAB9a**

(**A**) Genetic mapping of callus types with GBS segment markers on chromosome 9. The gray dash line indicates the LOD threshold of 3. (**B**) Genetic mapping of callus types from BSR-seq. The orange dash line indicates the threshold defined by the Bonferroni correction at the 5% significance level. The significance SNP markers are colored in green. The vertical purple dash lines (in both **A** and **B**) indicate the LOD support QTL interval, and the red vertical dash line indicates the left flanking of the interval adjusted based on the BSR-seq mapping. (**C**) The distribution of genotypes of the 60 XT-I F2 calli and 58 XT-II F2 calli on chromosome 9. The red rectangle box indicates the QTL interval, and the red triangle points at the QTL peak. (**D**) Distribution of callus types in three genotypes. AA: A188 homozygous genotype; BB: B73 homozygous genotype; AB: heterozygous genotype.

Figure S4. Effect of QTL markers on chromosomes 2, 5, 6, 8, and 9

(**A**, **B**, **C**, **D**, **E**) Effects of three genotypes at the QTLs of ctAB2a, ctAB5a, ctAB6a, ctAB8a, and ctAB9a. The y-axis represents the phenotype, and the x-axis represents different genotypes. Type I phenotype was coded as 1, and Type II was coded as number 2. Each open circle stands for an F2 individual callus. Numbers represent counts of individual calli in each group. The phenotype means of each genotype group were plotted in blue, purple and red with standard deviations. AA: A188 homozygous genotype; AB: heterozygous genotype; and BB: B73 homozygous genotype.

**Figure S5. The distribution of genotypes on chromosome 3**

The distribution of genotypes of the 60 XT-I F2 calli and 58 XT-II F2 calli on chromosome 2. The red rectangle box points at the genomic region that confers tissue culture response, of which the A188 allele contributes to a favorable tissue culture response.


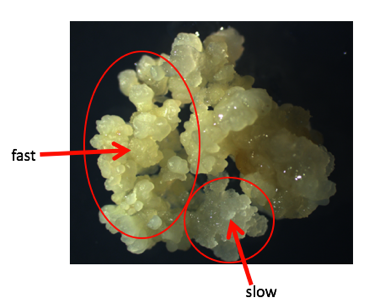


Figure S6. Fast- and slow-growing sections from a A188 callus

Fast- and slow-growing callus sections are highlighted using ovals and arrows.


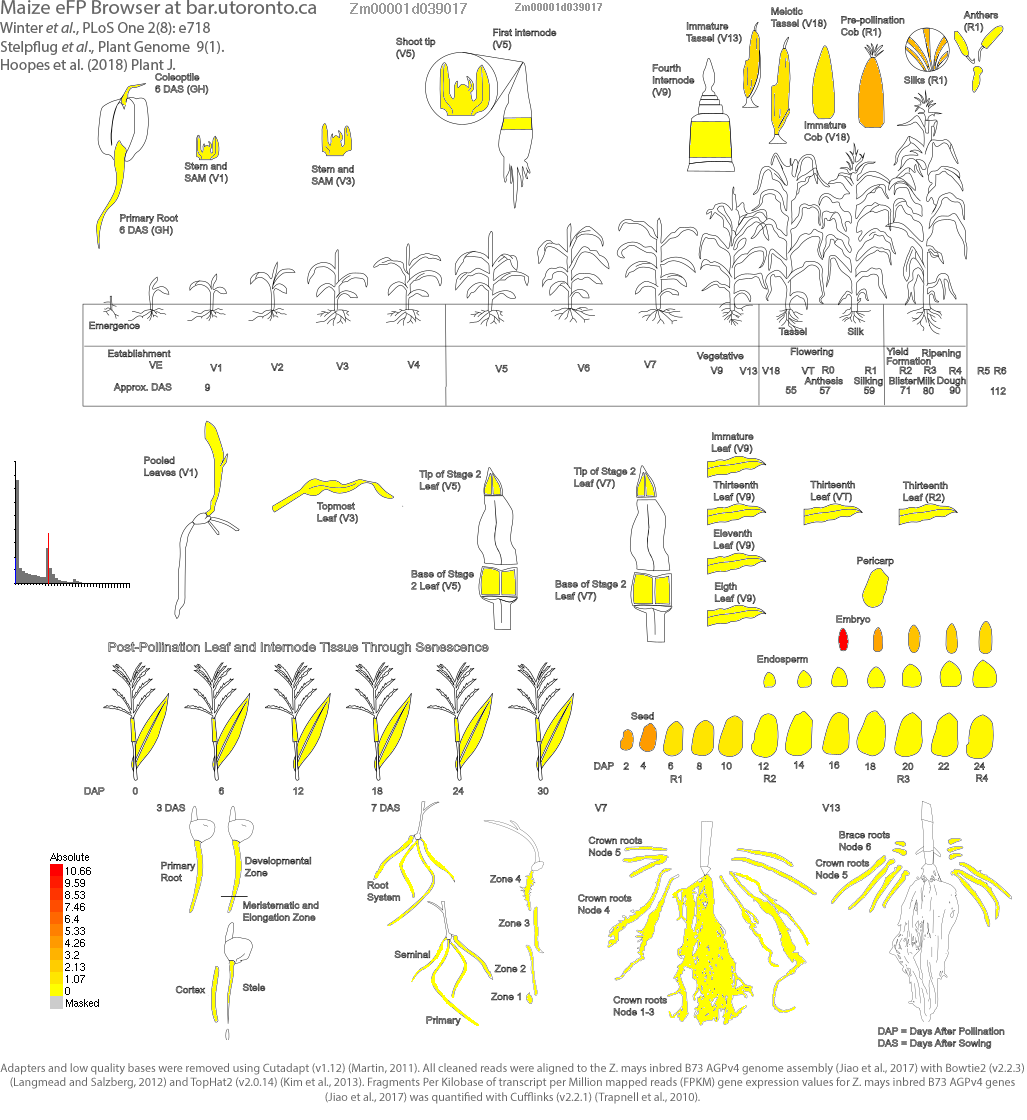


**Figure S7. B73 expression atlas of Zm00001d039017 (*wox9c*) from MaizeGDB.org**


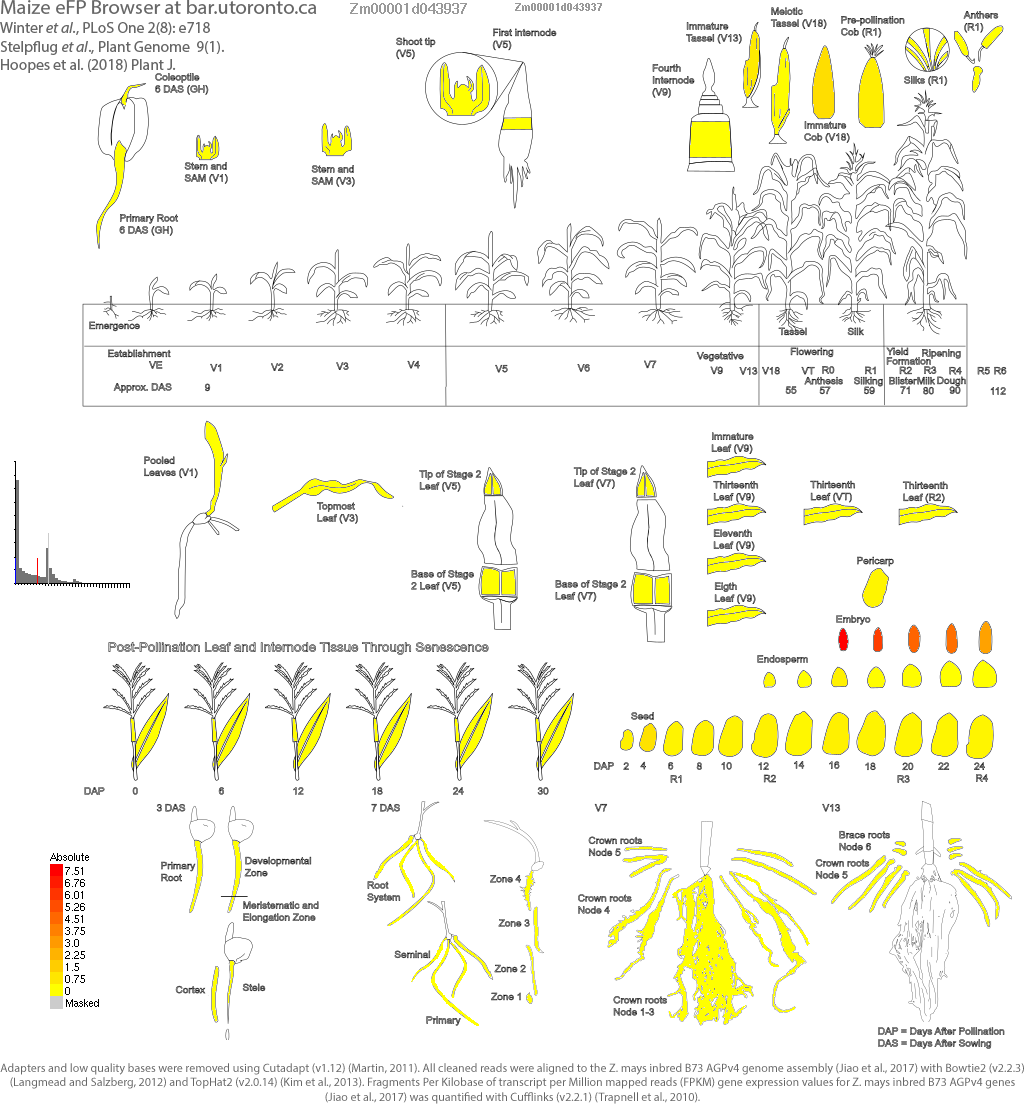


**Figure S8. B73 expression atlas of Zm00001d043937 (*wox9b*) from MaizeGDB.org**


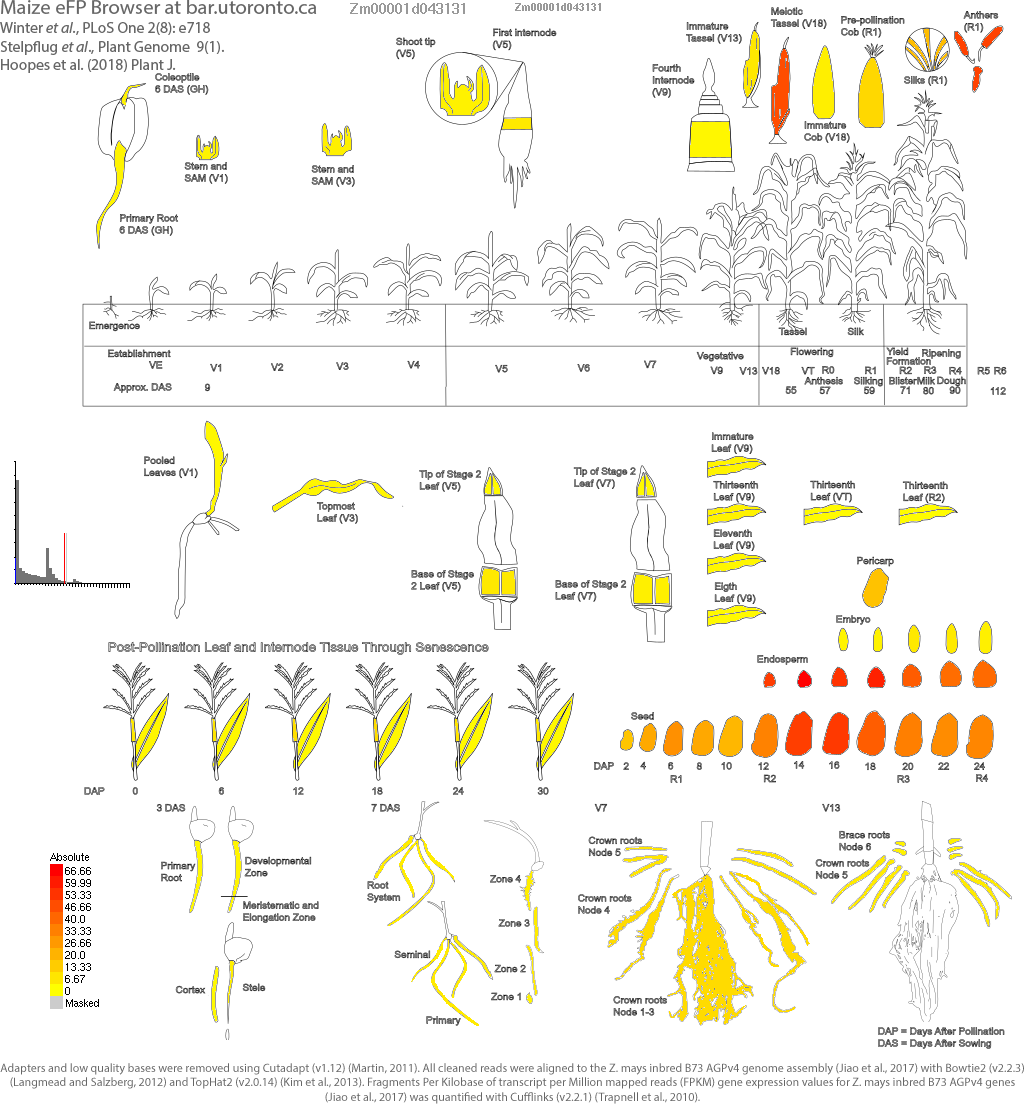


**Figure S9. B73 expression atlas of Zm00001d043131 (*myb138*) from MaizeGDB.org**


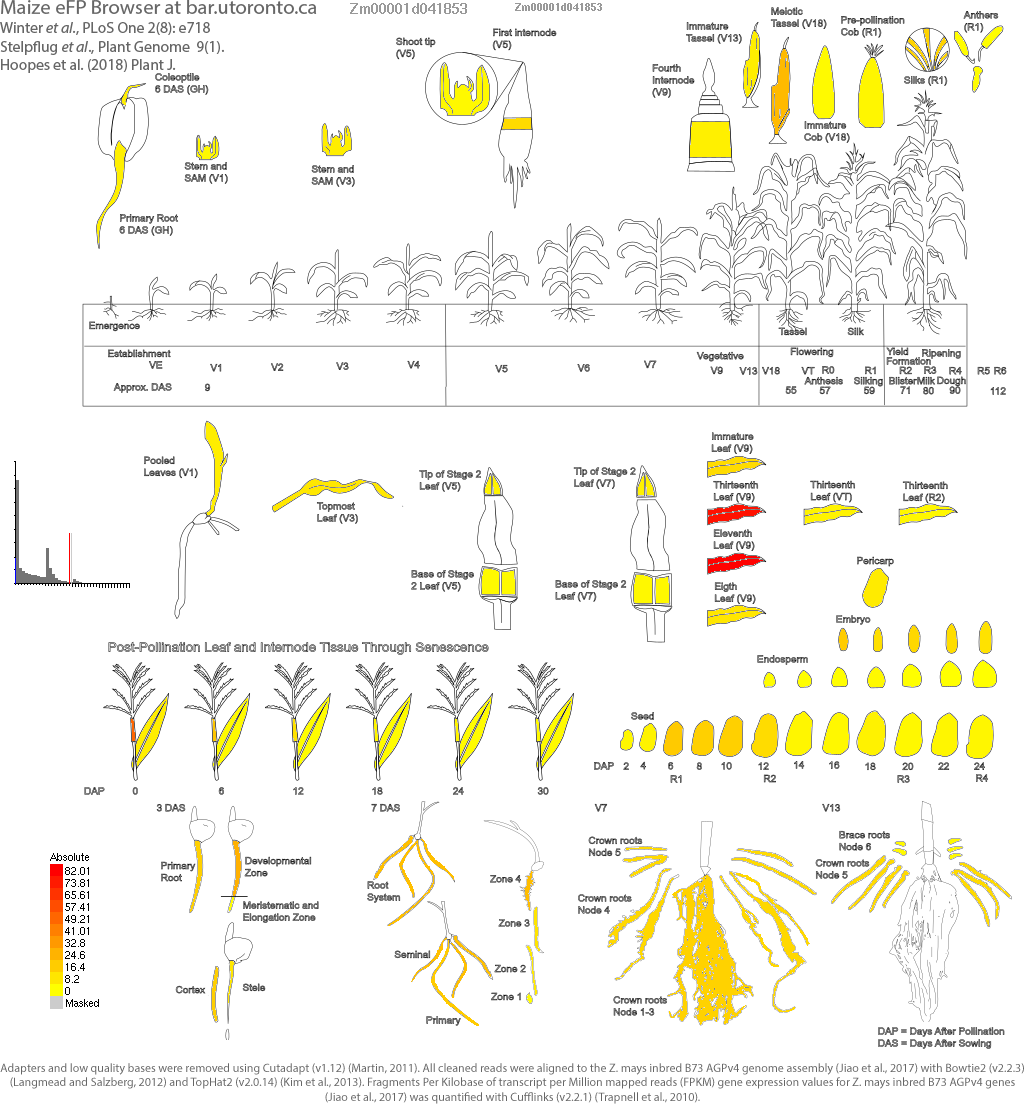


**Figure S10. B73 expression atlas of Zm00001d041853 (*myb8*) from MaizeGDB.org**


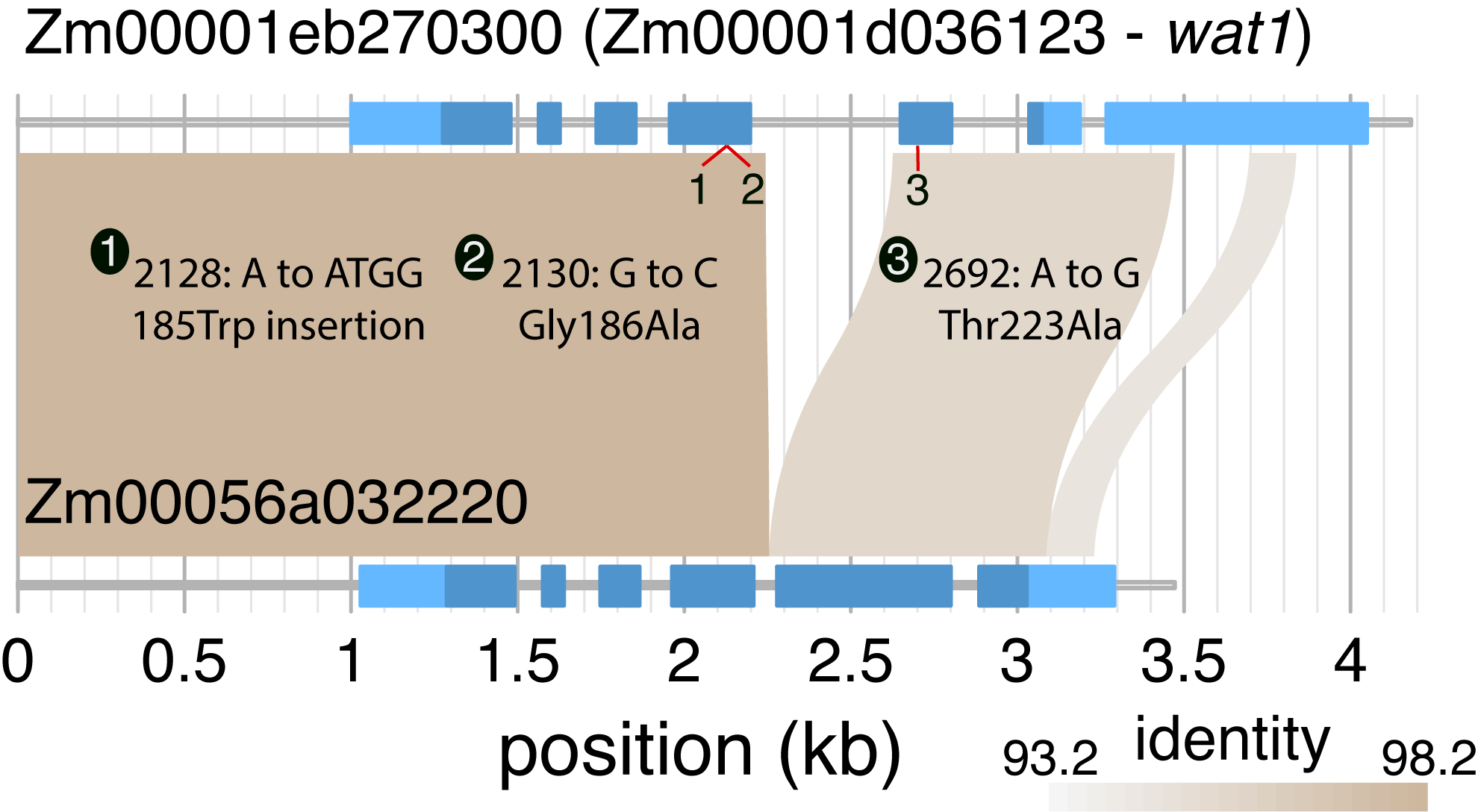


**Figure S11. Structural comparison of *wat1* alleles**

The B73 allele (Zm00001d036123) was compared with the A188 allele (Zm0056a032220) of *wat1*. Large insertion/deletion was identified in the fourth intron and the 3’ untranslated region (3’ UTR). In addition, three polymorphisms (labeled as 1, 2, 3) altering the protein product occur on the fourth and fifth exons.
